# Exosomes from adipose‐derived mesenchymal stem cells alleviate liver ischaemia reperfusion injury subsequent to hepatectomy in rats by regulating mitochondrial dynamics and biogenesis

**DOI:** 10.1111/jcmm.16952

**Published:** 2021-10-05

**Authors:** Qianzhen Zhang, Chenxi Piao, Haiyang Ma, Jiayuan Xu, Yue Wang, Tao Liu, Guodong Liu, Hongbin Wang

**Affiliations:** ^1^ College of Veterinary Medicine Northeast Agricultural University Harbin China; ^2^ College of Animal Science and Technology Jilin Agricultural University Changchun China

**Keywords:** adipose derived mesenchymal stem cells, exosomes, hepatectomy, ischaemia reperfusion, mitochondrial biogenesis, mitochondrial dynamics

## Abstract

Hepatic ischaemia reperfusion injury (HIRI) is a major factor leading to liver dysfunction after liver resection and liver transplantation. Adipose‐derived mesenchymal stem cells (ADSCs) have potential therapeutic effects on HIRI. Exosomes derived from ADSCs (ADSCs‐exo) have been widely studied as an alternative of ADSCs therapy. Thus, the aim of this study was to evaluate the potential protective effect and related mechanism of ADSCs‐exo on HIRI subsequent to hepatectomy. Rats were randomly divided into four groups: Sham, I30R+PH, ADSCs and ADSCs‐exo group. After 24 h of reperfusion, liver and serum of the rats were immediately collected. ADSCs‐exo improved liver function, inhibited oxidative stress and reduced apoptosis of hepatocytes in HIRI subsequent to hepatectomy in rats. ADSCs‐exo significantly promoted the recovery of mitochondrial function, markedly increased the content of ATP in the liver tissue, and improved the ultrastructure of mitochondria in hepatocytes. Moreover, ADSCs‐exo significantly increased the expression of OPA‐1, MFN‐1 and MFN‐2 proteins related to mitochondrial fusion, while DRP‐1 and Fis‐1 mRNA and protein expression associated with mitochondrial fission were significantly decreased after the treatment with ADSCs‐exo. In addition, ADSCs‐exo significantly increased the expression of PGC‐1α, NRF‐1 and TFAM genes and proteins related to mitochondrial biogenesis. ADSCs‐exo improves liver function induced by HIRI subsequent to hepatectomy in rats and maintains mitochondrial homeostasis by inhibiting mitochondrial fission, promoting mitochondrial fusion and promoting mitochondrial biogenesis. Therefore, ADSCs‐exo may be considered as a potential promising alternative to ADSCs in the treatment of HIRI subsequent to hepatectomy.

## 1 INTRODUCTION

Hepatic ischaemia reperfusion injury (HIRI) is the main factor contributing to liver dysfunction after liver resection and liver transplantation, which may further aggravate the liver surgical injury and increase postoperative morbidity and mortality.^1^ How to alleviate HIRI is an urgent problem to be solved in the medical research, and novel treatments are urgently required. Hepatic ischaemia results in disruption of energy supply to hepatocytes, leading to the death of hepatocytes.^2^ During reperfusion, a large amount of reactive oxygen species (ROS) and inflammatory factors further aggravate HIRI, promoting the occurrence of hepatocyte necrosis and apoptosis.^3^

The liver needs plenty of mitochondria to provide sufficient energy for its multiple physiological functions, since they are the major organelles responsible for the ATP generation through oxidative phosphorylation and for the maintenance of the homeostasis in the cellular environment. Recently, several studies confirmed that mitochondrial damage is one of the main factors leading to liver dysfunction in HIRI and fluoride‐induced liver injury.^4^, ^5^ ROS induces mitochondrial oxidative damage and reduce the production of ATP in mitochondria. Disruption of mitochondrial homeostasis causes mitochondrial fragmentation, leading to an inflammatory response that further aggravates ischaemia reperfusion injury.^6^, ^7^, ^8^ Mitochondrial biogenesis is involved in mitochondrial quality control and must be well coordinated to properly regulate cellular adaptation in response to mitochondrial damage.^9^ Mitochondrial dynamics regulates mitochondrial distribution and morphology through fusion and fission processes.^10^ Interventions targeting factors involved in mitochondrial homeostasis yielded beneficial results in the treatment of HIRI. Therefore, the reduction of mitochondrial injury may represent an ideal strategy to protect against HIRI.

Mesenchymal stem cells (MSCs) are adherent fibroblast‐like pluripotent cells possessing self‐renewal ability and multi‐directional differentiation potential under a specific environment, thus, they are widely investigated in the research of regenerative medicine and gene‐based therapy.^11^, ^12^ The advantages of adipose derived mesenchymal stem cells (ADSCs) include abundant sources, ease of obtaining, fat collection less harmful for the body, low immunogenicity and strong proliferation ability in vitro culture.^13^ ADSCs can prevent ischaemia reperfusion injury in the kidney, liver, heart and brain.^14^, ^15^, ^16^, ^17^ Studies indicated that MSCs perform biological functions and therapeutic effects on adjacent cells mainly by a paracrine mechanism.^18^, ^19^

Exosomes, with a diameter of 30–150 nm, are released by cells into the extracellular environment, and they represent the main bioactive vesicles of MSCs acting on surrounding cells.^20^, ^21^ MSCs‐derived exosomes have a protective effect on liver fibrosis, HIRI and drug‐induced liver injury.^22^, ^23^, ^24^ A previous study showed that MSCs protect against retinal ischaemia by improving mitochondrial function.^25^ However, it is not yet clear whether ADSCs‐exosomes (ADSCs‐exo) possesses the ability to ameliorate HIRI subsequent to hepatectomy. Therefore, the present study investigated the protective effect of ADSCs‐exo on HIRI subsequent to hepatectomy in a rat model and explored the mechanism of action of ADSCs‐exo on mitochondrial dynamics and mitochondrial biogenesis.

## 2 MATERIALS AND METHODS

### 2.1 Animals

Male Sprague‐Dawley rats (6–8 weeks, weighing 200–250 g) were purchased from Liaoning Changsheng biotechnology. All rats were fed with a unified standard chow, had free access to food and drinking water and were housed under standard conditions. All experimental protocols were approved by the Institutional Animal Care and Use Committee of the Northeast Agricultural University (NEAUEC 2019 05 08).

### 2.2 Experimental protocol

Twenty‐four rats were randomly divided into four groups and treated as follows:

1. Sham group (*n* = 6): Only an incision was made in the midline of the abdomen, and no clip was placed on the portal triad of the left and median lobes. Rats were treated with an intravenous injection of 600 μl PBS through the tail vein.
2. I30R+PH group (*n* = 6): A microvascular clip was placed on the portal triad of the left and median lobes to produce a 70% ischaemia for 30 min, and the left lobe was removed. Rats were treated with an intravenous injection of 600 μl PBS through the tail vein.
3. ADSCs group (*n* = 6): Rats were subjected to the same surgery as I30R+PH group. After surgery, they were treated with an intravenous injection of 2 × 10^6^ ADSCs in 600 μl PBS through the tail vein.
4. ADSCs‐exo group (*n* = 6): Rats were subjected to the same surgery as I30R+PH group. After surgery, they were treated with an intravenous injection of 100 μg ADSCs‐exo in 600 μl PBS through the tail vein.

The median lobe and blood samples were collected at 24 h after reperfusion in each group.

### 2.3 Surgical procedure

After a 12 h fast, rats were anaesthetized by isoflurane inhalation. A midline abdominal incision was performed to expose the portal triad of the left and median liver lobes, which was occluded with a noninvasive microvascular clamp for 30 min, resulting in 70% hepatic ischaemia, while the remaining 30% blood supply to the hepatic lobes prevented the congestion in the portal vein and gastrointestinal tract. The left hepatic lobe was ligated using a 3/0 silk thread and resected during the blockage of the blood flow.

### 2.4 Isolation and culture of ADSCs

The isolation and culture of ADSCs were performed as previously described.^15^ Briefly, the skin of the rats was incised and separated from the subcutaneous groyne fat to isolate the adipose tissue. The adipose tissue was washed three times with PBS, cut into small pieces and digested in PBS containing 1 mg/ml (0.1%) collagenase type I under gentle agitation at 37°C for 50 min, and subsequently centrifuged at 300 × *g* for 10 min. The undigested adipose tissue was removed and an equal volume of L‐DMEM (HyClone) containing 10% foetal bovine serum (FBS, Clark) was added to terminate the digestion. The mixture was filtered through a 75 μm cell strainer to remove additional undigested tissue fragments and centrifuged at 300 × *g* for 5 min. Then, the pellet was collected and incubated with an erythrocyte lysate for 5 min. The collected ADSCs were seeded in flasks containing L‐DMEM supplemented with 10% FBS, 2 mM L‐glutamine, 100 u/ml penicillin and 100 μg/ml streptomycin and incubated in a humidified incubator (Galaxy 170S, Eppendorf) at 37°C under 5% CO_2_. ADSCs were used and the phenotype was evaluated at passage 3–5.

### 2.5 Identification of ADSCs

Adipose‐derived mesenchymal stem cells at the passage 3–5 were characterized via osteogenic and adipogenic differentiation using differentiation media (Cyagen Biosciences). The adipogenic differentiation of the cells was evaluated after 2 weeks by the staining with 0.5% Oil Red O. The osteogenic differentiation of the cells was evaluated after 3 weeks by the staining with 0.1 mg/ml Alizarin Red.

The phenotype of ADSCs at the passage 3–5 was characterized by flow cytometry, resulting positive for CD29, CD44 and CD90, but not for CD45 (Biolegend). ADSCs were collected and digested using trypsin and washed twice in PBS. Then, they were incubated with antibodies (FITC‐CD29, 1:50; FITC‐CD44, 1:200; FITC‐CD45, 1:200; FITC‐CD90, 1:1000) on ice for 15–20 min in the dark. Next, they were washed twice, suspended in PBS, acquired by flow cytometry and analysed by FACSDiva software (BD Bioscience).

### 2.6 Preparation of ADSCs‐exo

Adipose‐derived mesenchymal stem cells at the passage 3–5 were cultured until reaching 80% confluence, and then, they were washed three times with PBS. The serum‐containing medium was replaced with serum‐free medium, and the cells were cultured for other 24 h. Then the supernatant was collected and subject to sequential centrifugation: 300 × *g* for 15 min, 2000 × *g* for 25 min, filtered through a 0.22 μm filter and centrifuged again at 10,000 × *g* for 50 min. The supernatant was transferred into a new ultracentrifugation tube (Beckman, 355618) and centrifuged at 100,000 × *g* for 90 min using a Ti‐50.2 rotor (Beckman). The pellet was resuspended in PBS and centrifuged at 100,000 × *g* for 90 min. Finally, the pellet containing exosomes was resuspended in PBS and stored at −80°C until further use.

### 2.7 Identification of ADSCs‐exo

The size of ADSCs‐exo was measured by nanoparticle tracking analysis (NTA) with ZetaView PMX110 (Particle Metrix). The morphology of ADSCs‐exo was analysed by transmission electron microscopy (TEM) H‐7650 (Hitachi). Western blot was performed using anti‐CD9 (Abcam, ab92726, 1:2000 dilution), anti‐CD81 (Santa, sc‐166029, 1:500 dilution) and anti‐TSG 101 (Abcam, ab125011, 1:5000 dilution).

### 2.8 Measurement of liver function and ATP content

Blood samples were centrifuged at 1800 × *g* for 15 min to collect the serum that was stored at −80°C until further use. The levels of serum alanine aminotransferase (ALT), aspartate aminotransferase (AST), total bilirubin (TBIL), alkaline phosphatase (ALP), total protein (TP) and lactate dehydrogenase (LDH) were determined using an automatic biochemical analyzer (BioTek). The ATP content in the liver tissue was measured using an ATP assay kit (Beyotime Biotechnology) according to the manufacturers' instructions.

### 2.9 Determination of oxidative stress

The 10% liver tissue homogenate solution was obtained by homogenization in cold normal saline and consequent centrifugation at 600 × *g* for 10 min at 4°C. The obtained supernatant was used to measure malonaldehyde (MDA), superoxide dismutase (SOD), glutathione peroxidase (GSH‐px) and catalase (CAT) using the corresponding assay kits from Nanjing Jiancheng Bioengineering Institute, Nanjing, China. The ROS level in the liver tissue was detected by DHE staining.

### 2.10 Detection of Caspase‐3 and Caspase‐9 activities

Liver tissue was homogenized using a lysis buffer included into the Caspase‐3 and Caspase‐9 activity assay kit (Beyotime). The supernatants were used to detect their activity according to the manufacturer's instructions. Protein concentration was determined using the Bradford assay kit (Beyotime).

### 2.11 TUNEL staining

The median lobe of the liver was fixed in 4% paraformaldehyde and embedded in paraffin using standard methods. The paraffin block was then cut into 5‐μm sections, which were dewaxed and rehydrated. Apoptotic cells were detected using the In Situ Cell Death Fluorescein Detection Kit (Roche Molecular Biochemicals) according to the manufacturer's instructions. The images were captured using a Nikon Ti‐U microscope.

### 2.12 Real‐time quantitative PCR (RT‐qPCR)

The total RNA from liver tissue was extracted using Trizol reagent (Invitrogen) and converted into cDNA using the PrimeScript RT reagent kit with gDNA Eraser (Takara) according to the manufacturer's protocol. The RT‐qPCR was performed using a LightCycler 480 I (Roche) following to the manufacturer's instruction. Relative mRNA expression was calculated by 2^−ΔΔCt^ method and normalized to β‐actin. The primer sequences were designed and synthesized by Sangon Biotech and listed in Table 1.

**TABLE 1:** Gene‐specific primers used for RT‐qPCR

Gene	Primer sequence
Bax	Forward 5′‐GACGCATCCACCAAGAAGCTGAG‐3′ Reverse 5′‐GCTGCCACACGGAAGAAGACC‐3′
Bcl‐2	Forward 5′‐AGCATGCGACCTCTGTTTGA‐3′ Reverse 5′‐TCACTTGTGGCCCAGGTATG‐3′
DRP‐1	Forward 5′‐ACAACAGGAGAAGAAAATGGAGT‐3′ Reverse 5′‐CGTTGGGCGAGAAAACCTTG‐3′
Fis‐1	Forward 5′‐GAATACGCCTGGTGCCTGGTTC‐3′ Reverse 5′‐GAAGACATAATCCCGCTGCTCCTC‐3′
OPA‐1	Forward 5′‐ATTTCGCTCCTGACCTGGAC‐3′ Reverse 5′‐GGTGTACCCGCAGTGAAGAA‐3′
MFN‐1	Forward 5′‐CGTGGCAGCAGCAGAGAAGAG‐3′ Reverse 5′‐CCTCCTCCGTGACCTCCTTGATC‐3′
MFN‐2	Forward 5′‐AGAGGCGATTTGAGGAGTGC‐3′ Reverse 5′‐CGCTCTTCCCGCATTTCAAG‐3′
PGC‐1α	Forward 5′‐CCTCACACCAAACCCACAGAGAAC‐3′ Reverse 5′‐TTGCGACTGCGGTTGTGTATGG‐3′
NRF‐1	Forward 5′‐TCTGCTGTGGCTGATGGAGAGG‐3′ Reverse 5′‐GATGCTTGCGTCGTCTGGATGG‐3′
TFAM	Forward 5′‐GCTAAACACCCAGATGCAAAA‐3′ Reverse 5′‐CGAGGTCTTTTTGGTTTTCC‐3′
β‐actin	Forward 5′‐TGTCACCAACTGGGACGATA‐3′ Reverse 5′‐GGGGTGTTGAAGGTCTCAAA‐3′

### 2.13 Western Blot analysis

Liver proteins were extracted by RIPA lysis buffer and western blot was performed as previously described.^26^ The primary antibodies used were the following: rabbit anti‐PGC‐1α (1:1000 dilution, wl02123, Wanlei); rabbit anti‐NRF1 (1:5000 dilution, ab175932, Abcam); rabbit anti‐TFAM (1:1000 dilution, ab252432, Abcam); rabbit anti‐MFN‐1 (1:1000 dilution, abs120025, absin); rabbit anti‐MFN‐2 (1:5000 dilution, ab124773, Abcam); rabbit anti‐OPA‐1 (1:3000 dilution, ab157457, Abcam); mouse anti‐DRP‐1 (1:500 dilution, sc‐271583, Santa Cruz); mouse anti‐Fis‐1 (1:500 dilution, sc‐376447, Santa Cruz); mouse anti‐Bax (1:500 dilution, sc‐23959, Santa Cruz); rabbit‐anti‐Bcl‐2 (1:500 dilution, wl01556, Wanlei) and rabbit anti‐β‐actin (1:5000 dilution, D110001, Sangon Biotech). The nitrocellulose membrane was incubated overnight at 4°C with the above primary antibodies and then incubated with the appropriate horseradish peroxidase (HRP)‐conjugated secondary antibodies at room temperature for 2 h. The bands were visualized using Tannon 5200 (Tanon Science & Technology Co., Ltd.) and quantified by Image J software (National Institutes of Health).

### 2.14 Statistical analysis

Statistical analysis was performed using SPSS 22.0 software. One‐way ANOVA was used for comparison among groups and LSD post analysis was performed. Results were presented as mean ± standard deviation. A value of *p* < 0.05 was considered statistically significant.

## 3 RESULTS

### 3.1 Characterization of ADSCs

**FIGURE 1:**
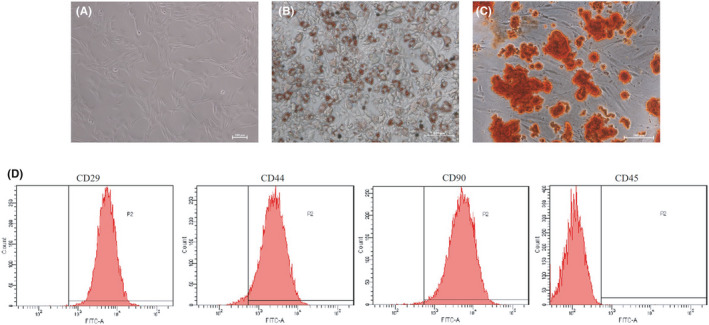
Characterization of ADSCs. (A) Passage three of ADSCs cultured in vitro (100× magnification). (B) Oil red O staining in ADSCs cultured in adipogenesis differentiation medium for 14 days (200× magnification). (C) Alizarin Red staining in ADSCs cultured in osteogenesis differentiation medium for 21 days (200× magnification). (D) Flow cytometry analysis of the surface markers in ADSCs

The primary ADSCs adhered to the culture flask after culturing them for 24 h. Most of the cells showed a typical spindle‐shaped cell body and proliferated rapidly. After 2–3 passages, all cells showed the typical fibroblast morphology, as shown Figure 1A. Oil red O staining (Figure 1B) and Alizarin red staining (Figure 1C) demonstrated their ability to differentiate into adipocytes and osteocytes, respectively. Flow cytometry analysis (Figure 1D) showed that these cells were positive for CD29 (99.9%), CD44 (97%) and CD90 (99.2%), but negative for CD45 (0.1%). These results revealed that ADSCs were successfully isolated from rat adipose tissue.

### 3.2 Identification of ADSCs‐exo

**FIGURE 2:**
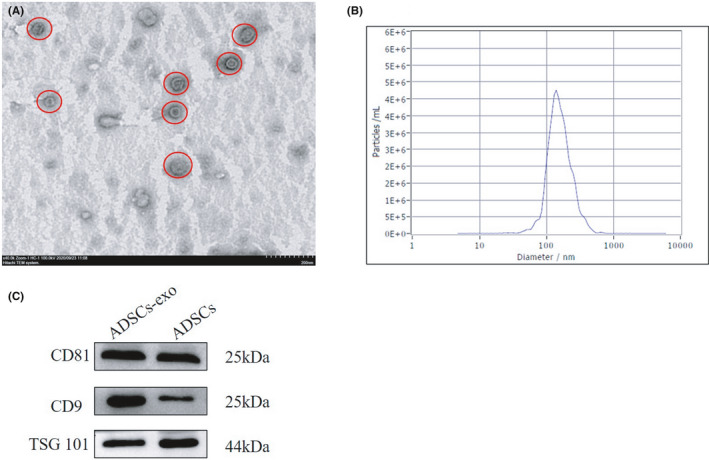
Identification of ADSCs‐exo. (A) Morphology of ADSCs‐exo by TEM (40,000× magnification). (B) Size distribution of ADSCs‐exo by Zeta sizer. (C) Western blot of CD81, CD9, and TSG101 protein expression in ADSCs‐exo

The exosomes were successfully isolated from the culture supernatant of ADSCs, and their typical cup‐shape morphology was observed by TEM (40,000×) (Figure 2A). Particle Metrix Analyzer showed that the average size of ADSCs‐exo was 142.3 nm (Figure 2B). Western blots results (Figure 2C) showed that the exosomal markers CD9, CD81 and TSG101 were expressed in exosomes. These results indicated that exosomes were successfully isolated from rat ADSCs.

### 3.3 Treatment with ADSCs‐exo improves liver function affected by I30R+PH

**FIGURE 3:**
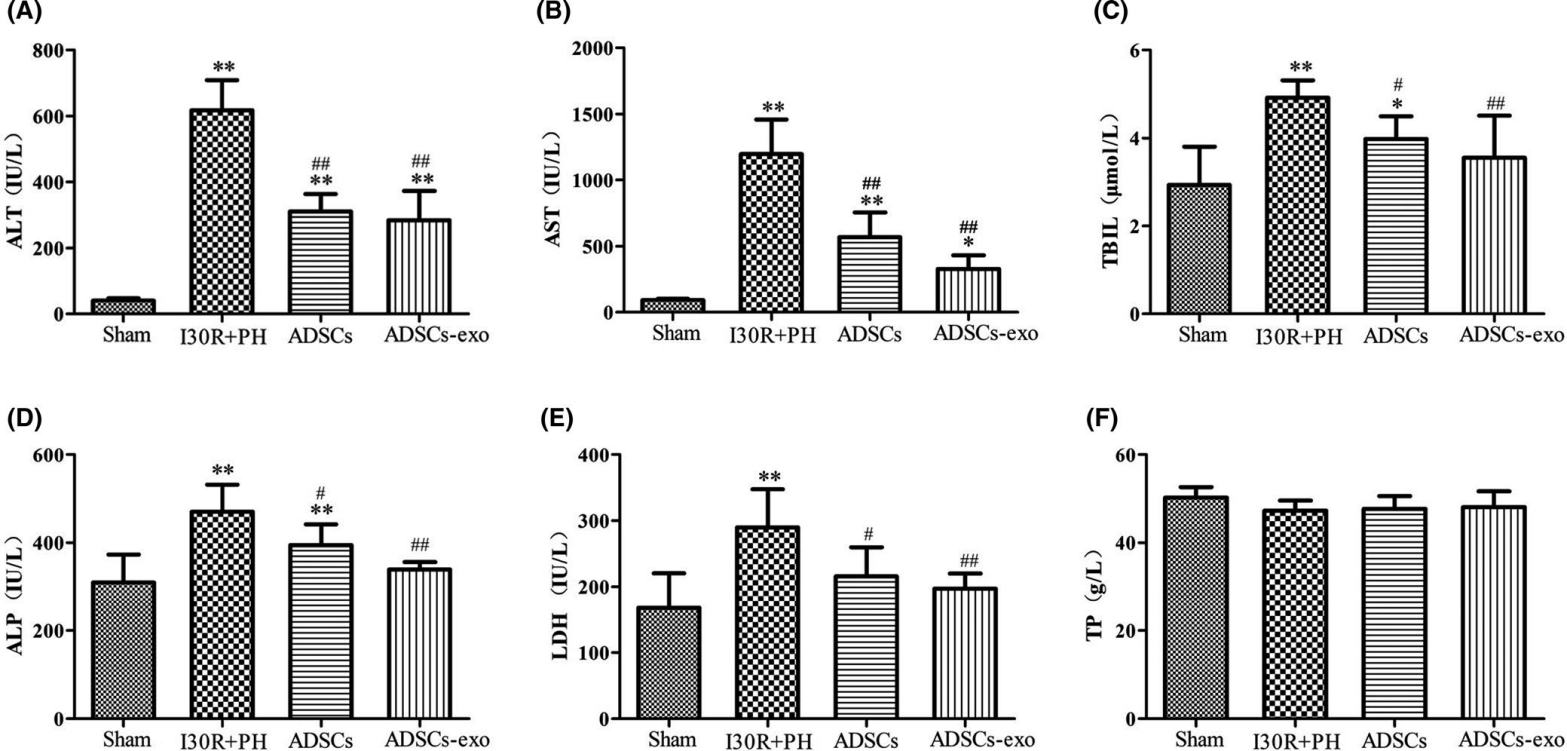
Treatment with ADSCs‐exo promotes the recovery of liver function after HIRI subsequent to hepatectomy. The serum levels of ALT (A), AST (B), TBIL (C), ALP (D), LDH (E) and TP (F) in each group, respectively. Results are presented as mean ± SD, ^*^ *p* < 0.05, ^**^ *p* < 0.01 versus the Sham group. ^#^ *p* < 0.05, ^##^ *p* < 0.01 versus the I30R+PH group

To clarify the effect of ADSCs‐exo on liver function after HIRI subsequent to hepatectomy in rats, serum ALT, AST, TBIL, ALP, LDH and TP levels were measured (Figure 3A–F). The levels of ALT (Figure 3A), AST (Figure 3B), TBIL (Figure 3C), ALP (Figure 3D) and LDH (Figure 3E) were significantly increased in I30R+PH group compared with the Sham group at 24 h (*p* < 0.01). ADSCs and ADSCs‐exo treatment remarkably decreased their levels in the serum (*p* < 0.01 or *p* < 0.05 vs. I30R+PH). However, no significant difference was observed in TP level among all the four groups (Figure 3F).

### 3.4 Treatment with ADSCs‐exo reduces oxidative stress due to I30R+PH

**FIGURE 4:**
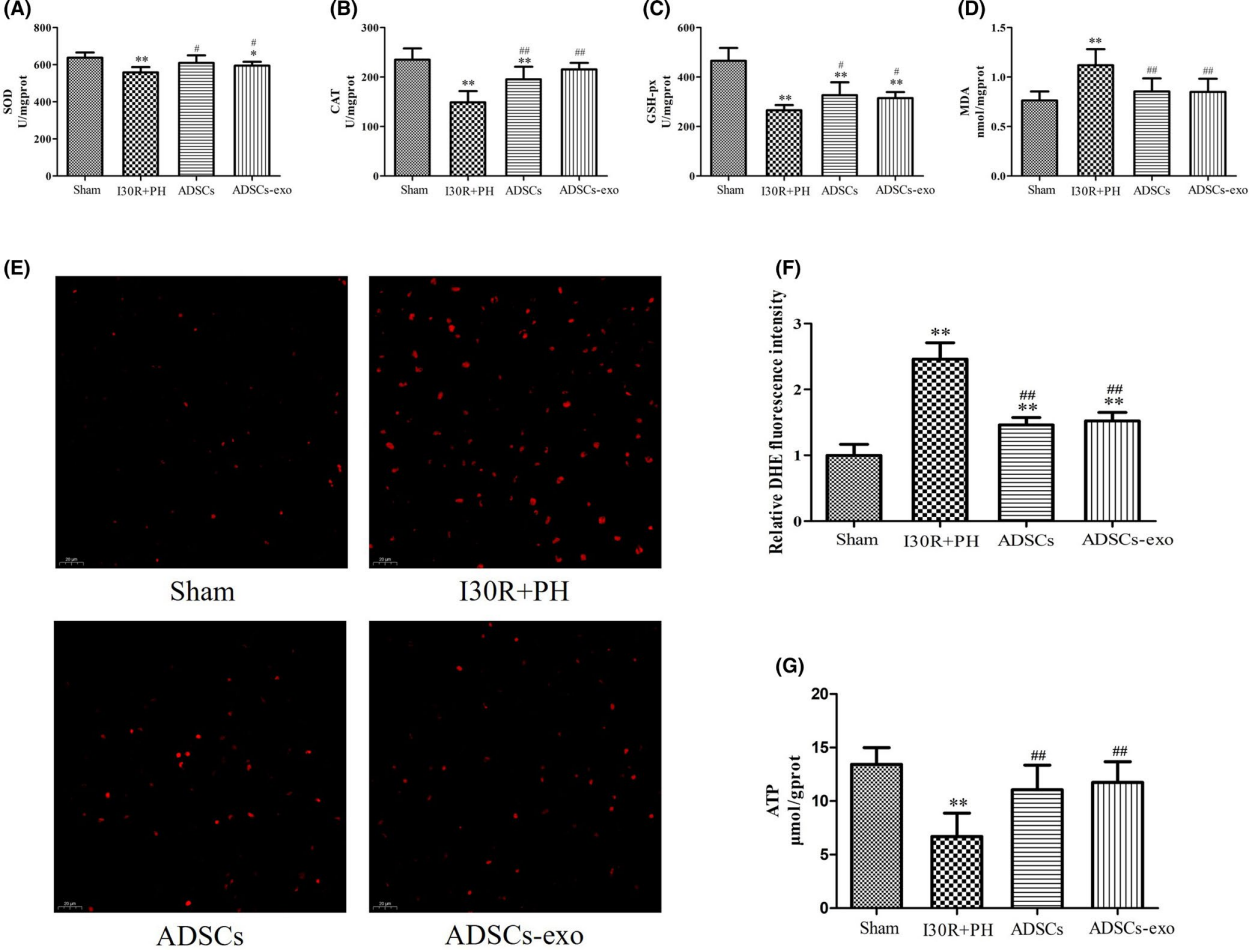
Treatment with ADSCs‐exo reduces oxidative stress due to HIRI subsequent to hepatectomy. The liver was harvested at 24 h after reperfusion. SOD (A), CAT (B), GSH‐px (C) and MDA (D) levels in the liver in each group. DHE (E) fluorescence staining (400× magnification) and relative quantification of the fluorescence intensity (F). ATP content in the liver of each group (G). Results are presented as mean ± SD, ^*^ *p* < 0.05, ^**^ *p* < 0.01 versus the Sham group. ^#^ *p* < 0.05, ^##^ *p* < 0.01 versus the I30R+PH group

The level of MDA in the liver of the I30R+PH group was significantly increased (*p* < 0.01) and SOD, CAT, GSH‐px levels were markedly reduced (*p* < 0.01) compared with their levels in the Sham group (Figure 4A–D). Administration of ADSCs and ADSCs‐exo significantly reduced the level of MDA in the liver (*p* < 0.01) and significantly increased the levels of SOD, CAT and GSH‐px compared to their level in the I30R+PH group (*p* < 0.01 or *p* < 0.05). The antioxidant effect of ADSCs and ADSCs‐exo in rats with HIRI subsequent to hepatectomy was also demonstrated (Figure 4E). Indeed, DHE fluorescence intensity (Figure 4F) in the I30R+PH group was significantly increased compared with that in the Sham group (*p* < 0.01). However, DHE fluorescence intensity was markedly reduced after the treatment with ADSCs and ADSCs‐exo compared with that in the I30R+PH group (*p* < 0.01).

### 3.5 Treatment with ADSCs‐exo increases ATP content after I30R+PH

The ATP content (Figure 4G) in the liver of the I30R+PH group was significantly reduced compared with its content in the Sham group (*p* < 0.01). However, its content significantly increased after ADSCs and ADSCs‐exo treatment (*p* < 0.05 vs. I30R+PH group).

### 3.6 Morphology of the liver tissue after treatment with ADSCs‐exo

**FIGURE 5:**
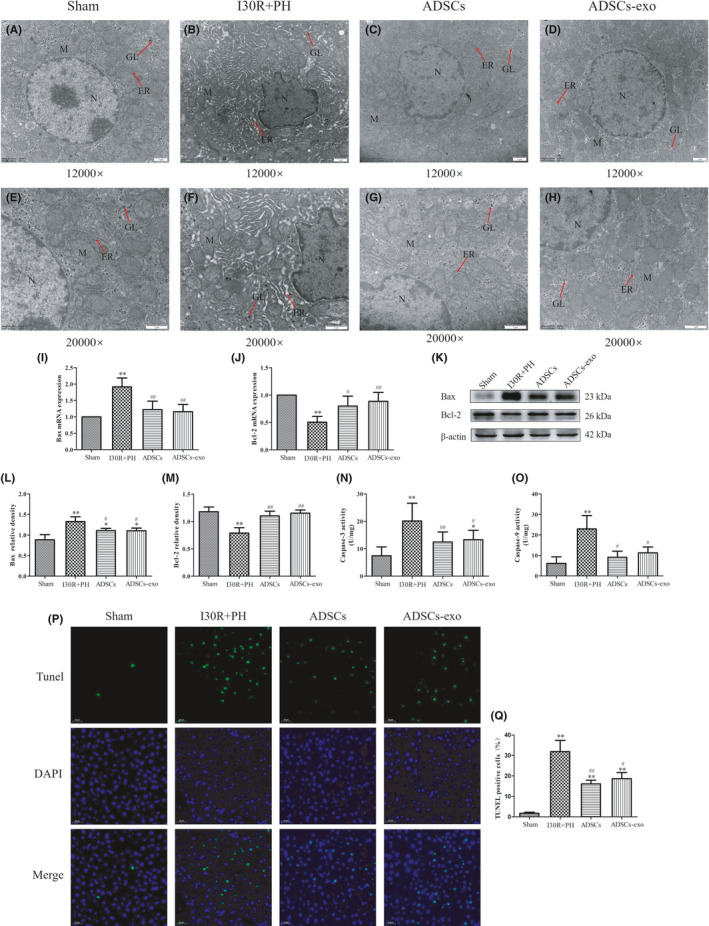
Effect of ADSCs‐exo on hepatocyte apoptosis after I30R+PH. (A–H) Ultrastructure of hepatocytes observed by TEM (12,000× and 20,000× magnification), N represents the nucleus; M represents mitochondria; ER represents the endoplasmic reticulum; and GL represents glycogen. Effect of ADSCs‐exo treatment on Bax (I) and Bcl‐2 (J) mRNA expression in the liver at 24 h after reperfusion. The expression of Bax and Bcl‐2 proteins in each group was analysed by Western blot (K). Quantification of Bax (L) and Bcl‐2 (M) in four groups. Effect of ADSCs‐exo on Caspase‐3 (N) and Caspase‐9 (O) activity in the liver. TUNEL staining (P) and the apoptotic rates (Q) in liver tissue. Results are presented as mean ± SD, ^*^ *p* < 0.05, ^**^ *p* < 0.01 versus the Sham group. ^#^ *p* < 0.05, ^##^ *p* < 0.01 versus the I30R+PH group

Transmission electron microscopy images showed a normal ultrastructure of the hepatocytes in the Sham group (Figure 5A,E). Nevertheless, hepatocytes from the I30R+PH group (Figure 5B,F) showed a severe ultrastructural damage with nuclear shrinkage, and a decrease in the number of mitochondria, which were also swollen. The endoplasmic reticulum in the I30R+PH group was severely expanded and the glycogen granules were decreased from the hepatocytes. Treatment with ADSCs (Figure 5C,G) and ADSCs‐exo (Figure 5D,H) alleviated the ultrastructural changes of hepatocytes observed above.

### 3.7 Treatment with ADSCs‐exo inhibits apoptosis due to I30R+PH

The expression of Bcl‐2 mRNA (Figure 5J) and protein (Figure 5K,M) in the I30R+PH group was markedly decreased when compared with the expression in the Sham group (*p* < 0.01). However, Bcl‐2 mRNA and protein expression in the ADSCs and ADSCs‐exo group were significantly increased when compared with their expression in the I30R+PH group (*p* < 0.01 or *p* < 0.05). The expression of Bax mRNA (Figure 5I) and protein (Figure 5K,L), as well as Caspase‐3 (Figure 5N) and Caspase‐9 activity (Figure 5O) in the I30R+PH group were significantly up‐regulated when compared to the Sham group (*p* < 0.01), while they were significantly down‐regulated after the treatment with ADSCs and ADSCs‐exo when compared with the I30R+PH group (*p* < 0.01 or *p* < 0.05). The apoptotic rate significantly increased in the I30R+PH group when compared with the rate in the Sham group (*p* < 0.01). However, the rate was remarkably reduced after ADSCs‐exo and ADSCs treatment (*p* < 0.01 or *p* < 0.05) (Figure 5P,Q).

### 3.8 Treatment with ADSCs‐exo inhibits excessive mitochondrial fission and promotes mitochondrial fusion after I30R+PH

**FIGURE 6:**
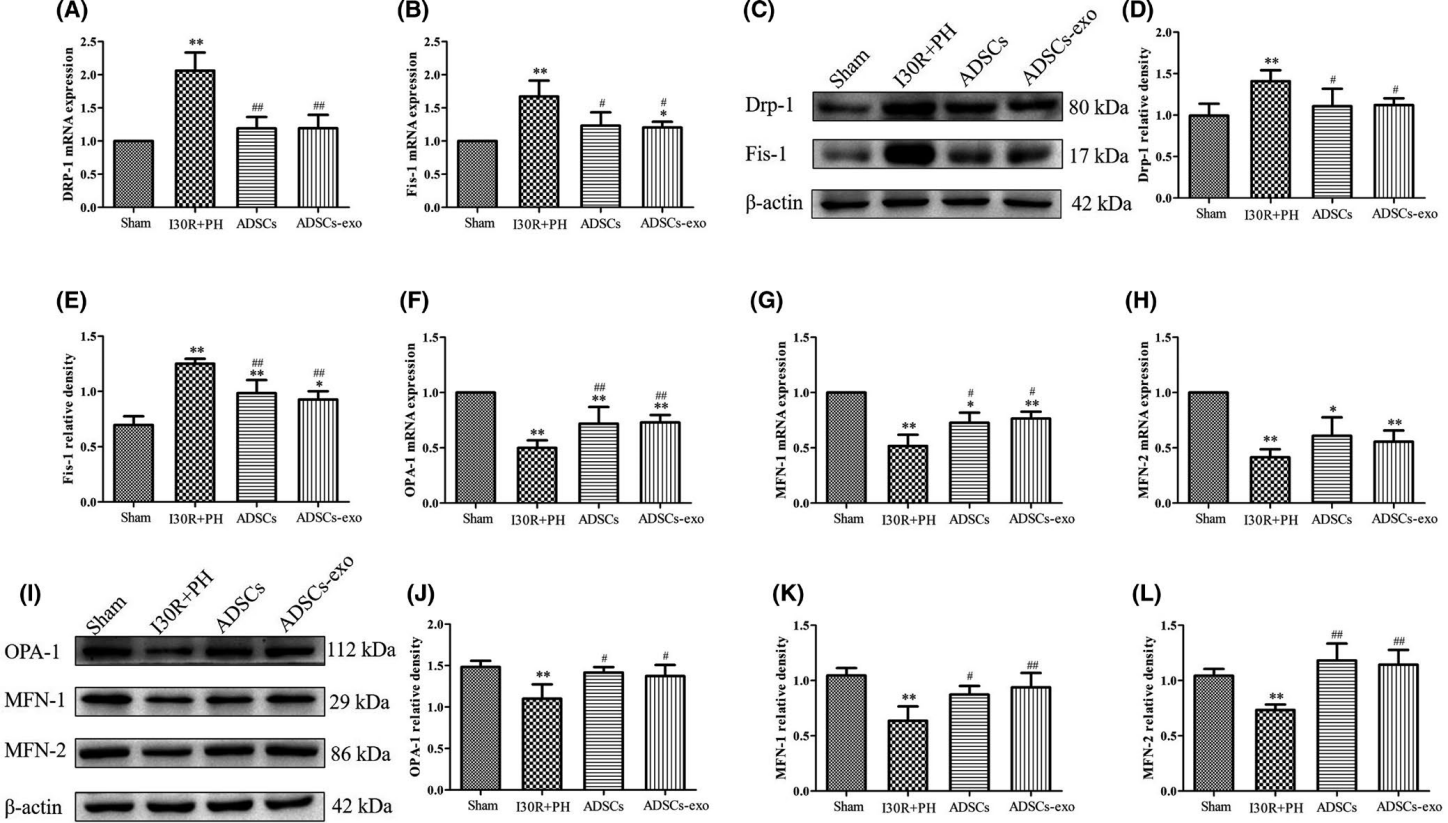
Effect of ADSCs‐exo on mitochondrial dynamics after HIRI subsequent to hepatectomy. The mRNA expression of genes related to mitochondrial dynamics such as DRP‐1 (A), Fis‐1 (B), OPA‐1 (F), MFN‐1 (G), MFN‐2 (H) by RT‐qPCR. DRP‐1, Fis‐1, OPA‐1, MFN‐1 and MFN‐2 protein expression in each group by Western blot (C and I). Quantification of DRP‐1 (D), Fis‐1 (E), OPA‐1 (J), MFN‐1 (K) and MFN‐2 (L) protein expression in four groups. Results are presented as mean ± SD, ^*^ *p* < 0.05, ^**^ *p* < 0.01 versus the Sham group. ^#^ *p* < 0.05, ^##^ *p* < 0.01 versus the I30R+PH group

The results indicated that DRP‐1 and Fis‐1 mRNA (Figure 6A,B) and protein expression (Figure 6C–E) were significantly increased in the I30R+PH group compared with their expression in the Sham group (*p* < 0.01). However, the treatment with ADSCs and ADSCs‐exo reduced their expression (*p* < 0.01 or *p* < 0.05 vs. I30R+PH group). The mRNA (Figure 6F–H) and protein (Figure 6I–L) expression of factors related to mitochondrial fusion, such as OPA‐1, MFN‐1 and MFN‐2 was significantly inhibited in the I30R+PH group compared with the Sham group (*p* < 0.01). Conversely, treatment with ADSCs and ADSCs‐exo significantly improved the expression of the above mentioned proteins (*p* < 0.01 or *p* < 0.05 vs. I30R+PH group). In addition, ADSCs and ADSCs‐exo significantly increased the mRNA expression of OPA‐1 and MFN‐1 (*p* < 0.01 or *p* < 0.05 vs. I30R+PH group), ADSCs and ADSCs‐exo increased the mRNA expression of MFN‐2, but no significantly difference compared with I30R+PH group (*p* > 0.05).

### 3.9 Treatment with ADSCs‐exo promotes mitochondrial biogenesis after I30R+PH

**FIGURE 7:**
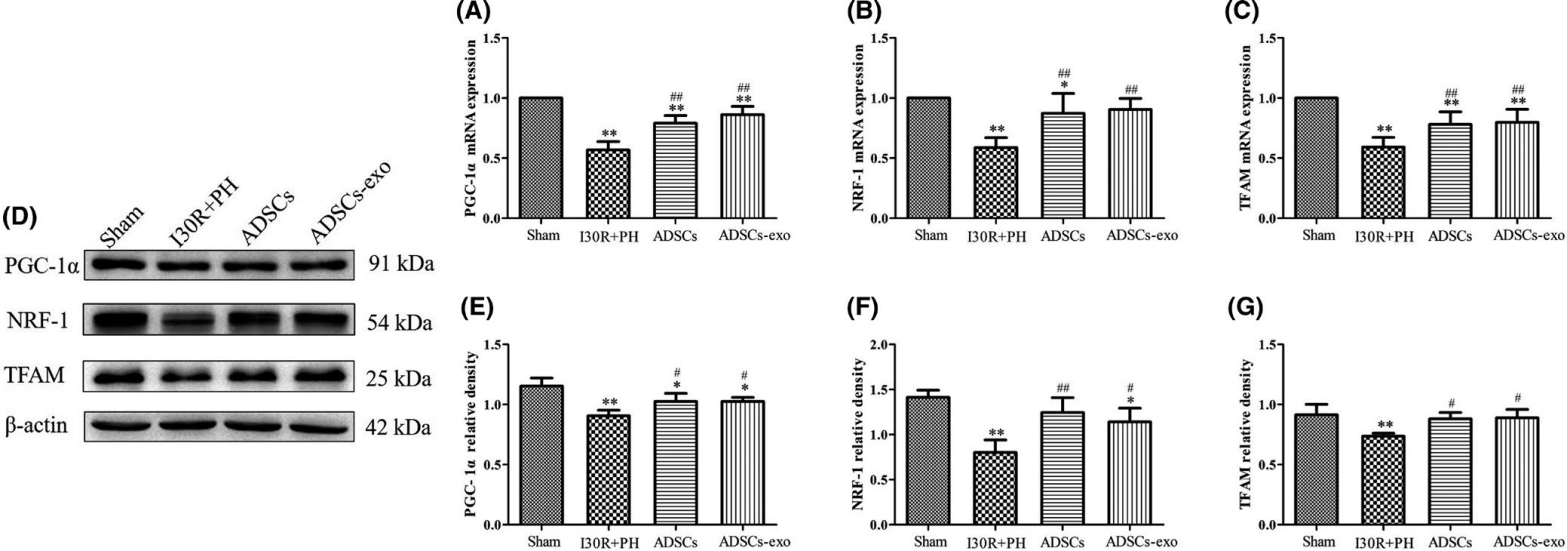
Effect of ADSCs‐exo on mitochondrial biogenesis after HIRI subsequent to hepatectomy. PGC‐1α (A), NRF‐1 (B) and TFAM (C) mRNA expression in each group by RT‐qPCR. PGC‐1α, NRF‐1 and TFAM protein expression in each group by Western blot (D). Quantification of PGC‐1α (E), NRF‐1 (F) and TFAM (G) protein expression in four groups. Results are presented as mean ± SD, ^*^ *p* < 0.05, ^**^ *p* < 0.01 versus the Sham group. ^#^ *p* < 0.05, ^##^ *p* < 0.01 versus the I30R+PH group

The expression of PGC‐1α, TFAM and NRF‐1 mRNA and protein were markedly decreased at 24 h after HIRI subsequent to hepatectomy (*p* < 0.01 vs. Sham group) (Figure 7A–G). However, both the above mRNA and protein expression were significantly increased in ADSCs and ADSCs‐exo group when compared with the I30R+PH group (*p* < 0.01 or *p* < 0.05).

## 4 DISCUSSION

The present study revealed at first that the treatment with ADSCs and ADSCs‐exo could significantly reduce HIRI subsequent to hepatectomy in rats. The potential mechanism of ADSCs‐exo might be related to the inhibition of apoptosis induced HIRI subsequent to hepatectomy. Our results further proved that ADSCs‐exo could maintain mitochondrial homeostasis and improve mitochondrial function by regulating mitochondrial dynamics and biogenesis. Thus, ADSCs‐exo might represent a potential promising cell‐free therapeutic method to combat liver injury.

Some studies found that the pathogenesis of HIRI mainly includes the overproduction of ROS, intracellular calcium overload, and inflammatory response. The continuous release of ROS disrupts the antioxidant system, leading to oxidative stress.^27^ Thus, ROS scavenging is essential for attenuating HIRI.^28^ A recent study confirmed that the ADSCs‐exo exert an antioxidant effect by suppressing ROS production.^29^ The present study found that ADSCs‐exo treatment significantly decreased the levels of oxidative damage indicators such as MDA and ROS in the liver, while markedly increased the content of antioxidant enzymes SOD, CAT and GSH‐px after HIRI subsequent to hepatectomy. Thus, the present results demonstrated that ADSCs‐exo protected against oxidative stress after HIRI subsequent to hepatectomy in rats.

The mechanism inducing hepatocyte death caused by HIRI typically involves apoptosis. Mitochondria are involved in metabolic and energy for cells, and regulate apoptosis. Mitochondrial injury caused by excessive ROS reduces mitochondrial membrane potential and down‐regulate ATP synthesis, subsequently leading to cell apoptosis.^30^ Ischaemia and hypoxia induce mitochondrial swelling and cytochrome C release into the cytoplasm, which further promotes the caspase cascade reaction and stimulates apoptosis.^31^ The regulation of hepatocyte apoptosis might provide a therapeutic method to cure HIRI. One study demonstrated that exosomes from human‐induced pluripotent stem cell‐derived mesenchymal stromal cells protect against liver injury and inhibit apoptosis in HIRI.^32^ Additionally, ADSCs‐exo protect H9c2 cells from H_2_O_2_ induced apoptosis and reduce apoptosis in cardiac ischaemia reperfusion injury.^33^ In the present study, the treatment with ADSCs‐exo decreased apoptosis by inhibiting the activity of Caspase‐3 and Caspase‐9, decreasing Bax mRNA and protein expression and increasing Bcl‐2 mRNA and protein expression. These results indicated that ADSCs‐exo inhibited apoptosis, thus, they were beneficial to alleviate HIRI subsequent to hepatectomy in rats.

Mitochondria homeostasis determines cell fate during ischaemia reperfusion injury.^34^ In this study, the morphology of hepatocytes revealed that the damage of mitochondria was significantly reduced after ADSCs‐exo treatment. As regard the ATP production our results were consistent with those of previous studies,^35^, ^36^ since the ATP content in the liver of the I30R+PH group was significantly lower than that in the Sham group, which might be due to a direct consequence of impaired mitochondrial dynamics. ADSCs‐exo treatment reversed the decrease of ATP content caused by HIRI subsequent to hepatectomy. Thus, our hypothesis was that mitochondrial injury and functional recovery might be associated with ADSCs‐exo regulation of mitochondrial dynamics balance.

Mitochondria are highly dynamic organelles that continuously undergo two opposite processes such as fission and fusion to maintain mitochondrial function under physiological conditions.^37^ Thus, mitochondrial fission and fusion are used to evaluate the morphology, quantity and abundance of mitochondria in liver diseases.^38^ Excessive mitochondrial fission induces mitochondrial fragmentation, eventually leading to apoptosis.^39^ Mitochondrial fission mainly involves the fission of the outer mitochondrial membrane, and DRP‐1 and Fis‐1 are proteins regulating it in mammalian cells.^40^ In HIRI, persistent metabolic stress promotes mitochondrial fragmentation through the overexpression of Fis‐1 and DRP‐1.^41^ In the current study, ADSCs‐exo treatment significantly decreased the expression of DRP‐1 and Fis‐1 after HIRI subsequent to hepatectomy. Mitochondrial fusion maintains the stability of mitochondrial membrane potential by the fusion with neighbouring depolarized mitochondria, and it is a process generally believed to be beneficial for maintaining mitochondrial function.^42^, ^43^, ^44^ MFN‐1 and MFN‐2 are the regulatory proteins of mitochondrial outer membrane fusion, while OPA‐1 mediates the fusion of mitochondrial inner membrane.^45^ In our present study ADSCs‐exo treatment significantly enhanced the expression of MFN‐1, MFN‐2 and OPA‐1. These results suggested that ADSCs‐exo might inhibit apoptosis by down‐regulating excessive mitochondrial fission and up‐regulating mitochondrial fusion.

One of the significant findings in this study was that the treatment with ADSCs‐exo promoted mitochondrial biogenesis after HIRI subsequent to hepatectomy in rats. Mitochondrial biogenesis is one of the mechanisms regulating the quality and quantity of mitochondria and PGC‐1α is regulating it. PGC‐1α promotes the increase of NRF‐1 expression and interacts with NRF‐1 to regulate mitochondrial biogenesis.^46^ Therefore, the induction of PGC‐1α expression in the liver is a critical procedure for the energy metabolism pathway for the production of ATP and for maintaining the homeostasis of the mitochondrial environment. NRF‐1 is a transcription factor that regulates five respiratory complexes synthesis and TFAM.^47^ TFAM is a transcription factor necessary to regulate the transcription and replication of mitochondrial DNA.^48^ However, persistent hypoxia and ischaemia impairs mitochondrial biogenesis and inhibits protein expression in the PGC‐1α‐NRF‐1‐TFAM pathway.^49^ Recent studies suggested that the enhancement of mitochondrial biogenesis protects against HIRI.^50^, ^51^ Our results showed that the expression of PGC‐1α, NRF‐1 and TFAM were significantly decreased after HIRI subsequent to hepatectomy, but these indicators were markedly increased after ADSCs‐exo treatment. These results indicated that ADSCs‐exo had the ability to enhance mitochondrial biogenesis and protected against mitochondrial dysfunction induced by HIRI subsequent to hepatectomy.

In summary, our study is the first demonstrating that ADSCs‐exo protects against HIRI subsequent to hepatectomy by reducing mitochondrial fission, promoting mitochondrial fusion and improving mitochondrial biogenesis. Thus, ADSCs‐exo treatment might be considered as a potential novel and promising therapeutic approach to combat HIRI subsequent to hepatectomy, providing a basis for the potential clinical application of ADSCs‐exo in liver surgery.

## CONFLICT OF INTEREST

The authors declare that there are no conflict of interest.

## AUTHOR CONTRIBUTION

**Qianzhen Zhang:** Conceptualization (lead); Data curation (lead); Investigation (lead); Methodology (lead); Project administration (lead); Software (lead); Writing‐original draft (lead); Writing‐review & editing (lead). **Chenxi Piao:** Investigation (equal); Methodology (equal); Software (equal); Writing‐review & editing (supporting). **Haiyang Ma:** Investigation (equal); Methodology (equal); Software (equal). **Jiayuan Xu:** Investigation (equal); Methodology (equal); Software (equal). **Yue Wang:** Investigation (equal); Writing‐review & editing (supporting). **Tao Liu:** Investigation (supporting); Methodology (supporting). **Guodong Liu:** Investigation (supporting); Methodology (supporting). **Hongbin Wang:** Conceptualization (equal); Funding acquisition (lead); Investigation (lead); Methodology (lead); Project administration (lead); Software (equal); Supervision (lead); Validation (lead); Writing‐review & editing (equal).

## Data Availability

The data that support the findings of this study are available from the corresponding author upon reasonable request.
